# Unravelling the complex story of intergenomic recombination in ABB allotriploid bananas

**DOI:** 10.1093/aob/mcaa032

**Published:** 2020-04-07

**Authors:** Alberto Cenci, Julie Sardos, Yann Hueber, Guillaume Martin, Catherine Breton, Nicolas Roux, Rony Swennen, Sebastien Christian Carpentier, Mathieu Rouard

**Affiliations:** 1 Alliance Bioversity International - CIAT, Montpellier, France; 2 AGAP, Université de Montpellier, CIRAD, INRA, Montpellier SupAgro, Montpellier, France; 3 CIRAD, UMR AGAP, Montpellier, France; 4 Alliance Bioversity International - CIAT, Leuven, Belgium; 5 Laboratory of Tropical Crop Improvement, Division of Crop Biotechnics, KU Leuven, Leuven, Belgium; 6 International Institute of Tropical Agriculture, c/o The Nelson Mandela African Institution of Science and Technology (NM-AIST), Arusha, Tanzania

**Keywords:** Genetic diversity, homologous exchanges, meiosis, *Musa*, polyploids, subgenomes

## Abstract

**Background and Aims:**

Bananas (*Musa* spp.) are a major staple food for hundreds of millions of people in developing countries. The cultivated varieties are seedless and parthenocarpic clones of which the ancestral origin remains to be clarified. The most important cultivars are triploids with an AAA, AAB or ABB genome constitution, with A and B genomes provided by *M. acuminata* and *M. balbisiana*, respectively. Previous studies suggested that inter-genome recombinations were relatively common in banana cultivars and that triploids were more likely to have passed through an intermediate hybrid. In this study, we investigated the chromosome structure within the ABB group, composed of starchy cooking bananas that play an important role in food security.

**Methods:**

Using SNP markers called from RADSeq data, we studied the chromosome structure of 36 ABB genotypes spanning defined taxonomic subgroups. To complement our understanding, we searched for similar events within nine AB hybrid genotypes.

**Key Results:**

Recurrent homologous exchanges (HEs), i.e. chromatin exchanges between A and B subgenomes, were unravelled with at least nine founding events (HE patterns) at the origin of ABB bananas prior to clonal diversification. Two independent founding events were found for Pisang Awak genotypes. Two HE patterns, corresponding to genotypes Pelipita and Klue Teparod, show an over-representation of B genome contribution. Three HE patterns mainly found in Indian accessions shared some recombined regions and two additional patterns did not correspond to any known subgroups.

**Conclusions:**

The discovery of the nine founding events allowed an investigation of the possible routes that led to the creation of the different subgroups, which resulted in new hypotheses. Based on our observations, we suggest different routes that gave rise to the current diversity in the ABB cultivars, routes involving primary AB hybrids, routes leading to shared HEs and routes leading to a B excess ratio. Genetic fluxes took place between *M. acuminata* and *M. balbisiana*, particularly in India, where these unbalanced AB hybrids and ABB allotriploids originated, and where cultivated *M. balbisiana* are abundant. The result of this study clarifies the classification of ABB cultivars, possibly leading to the revision of the classification of this subgroup.

## INTRODUCTION

Bananas (*Musa* spp.) are herbaceous monocotyledons belonging to the Zingiberales. The *Musa* genus originated in South-East Asia and west Oceania where it was domesticated; from there banana was spread to tropical areas of Africa and America. Two species contributed widely to the varieties cultivated worldwide: *Musa acuminata* Colla (A genome), whose distribution coincides with Mainland and Maritime South-East Asia and New Guinea island, and *M. balbisiana* Colla (B genome), distributed in eastern South Asia, northern South-East Asia and southern China (Simmonds, 1962; Janssens *et al.*, 2016).

Present-day cultivated bananas with an exclusively A genome constitution are noted as AA or AAA and hybrids are noted as AB, AAB or ABB, depending on their ploidy level. Diploids with an AA constitution are parthenocarpic with various degrees of sterility, mainly cultivated for fruit consumption. Edible AB hybrids do exist but are rare, and are mostly known in India. Those studied so far are reported as sterile (Jenny *et al.*, 2011). *Musa balbisiana*, although associated with wild populations, can be found in home gardens, where it is cultivated for its leaves, male buds, young fruits or seeds, the last being used in traditional pharmacopoeias (Subbaraya *et al.*, 2006). The most common banana cultivars are triploid (2*n* = 3*x* = 33), i.e. composed of three sets of 11 chromosomes, and are either autotriploid (AAA) or allotriploid (AAB and ABB). These cultivars have very low seed set due to farmers’ selection combined with triploidy, which hampers the production of balanced gametes. Consequently, they are clonally propagated. The combination of sterility and parthenocarpy ensures the production of seedless fruits that are edible.

The taxonomic classification of allotriploid bananas was mainly based on morphological descriptors with traits differentiating *M. acuminata* from *M. balbisiana* (Simmonds and Shepherd, 1955). Later, molecular analysis of organelle genomes allowed the characterization of cultivar cytotypes and, combined with ploidy measurements, led to hypotheses on crossing pathways (Carreel *et al.*, 2002; Boonruangrod *et al.*, 2008). Next, molecular markers from single sequence repeats (SSRs) provided a multilocus survey of the parental allele contribution (Hippolyte *et al.*, 2012; Christelová *et al.*, 2017). Cytogenetic and genome-wide studies also revealed that allotriploids are not mere additions of sets of 11 chromosomes, but contain regions with a variable subgenome ratio along the chromosomes (D’Hont *et al.*, 2000; Jeridi *et al.*, 2011; Noumbissié *et al.*, 2016), demonstrating the occurrence of recombinations between subgenomes. Advances in next-generation sequencing enabled the development of methods allowing (1) a more detailed view of the chromosome structure of a few allopolyploid cultivars (Wang *et al.*, 2019), (2) to follow up segregation of chromosomes on breeding materials (Baurens *et al.*, 2019) and (3) an understanding of the impact of allopolyploidy on gene expression (Cenci *et al.*, 2019).

It is commonly accepted that triploids have been generated by spontaneous hybridizations of edible AA or AB plants with various sources of the additional A or B genome (other edible AA or *M. acuminata*, and *M. balbisiana*). However, the studies cited above point up the genomic complexity of allotriploid cultivars, suggesting the occurrence of backcrosses with parental species through residual fertility in some allotriploid cultivars (De Langhe *et al.*, 2010).

Among the allotriploids, the ABB genomic group comprises starchy bananas that are used for cooking, dessert and beer production (Karamura *et al.*, 1998). They are reported as being resistant to weevils, nematodes and black leaf streak (Karamura *et al.*, 1998) and tolerant of drought (Thomas *et al.*, 1998; De Langhe, 2002; Vanhove *et al.*, 2012; Ravi *et al.*, 2013; Kissel *et al.*, 2015; van Wesemael *et al.*, 2019). The ABB banana cultivars belong to a morphologically diverse group subdivided into nine subgroups, namely Bluggoe, Monthan, Ney Mannan, Klue Teparod, Kalapua, Peyan, Pisang Awak, Pelipita and Saba (Daniells *et al.*, 2001). They originate from two regions: India and surrounding regions, and South-East Asia (De Langhe *et al.*, 2009; Perrier *et al.*, 2011).

Molecular markers have shown that some ABB cultivars were erratically classified (Sardos *et al.*, 2016; Christelová *et al.*, 2017). Furthermore, the genomic composition of the Saba subgroup, coming from the Philippines, has been debated for years as some authors have suggested it consists of B genomes only (Valmayor *et al.*, 1999).

The goals of this study were (1) to survey a large sample of interspecific AB and ABB genotypes to establish molecular karyotypes and thus to set up a clear framework for their classification, and (2) to bring new insights into the origin and evolution of ABB triploid cultivars.

## MATERIALS AND METHODS

### Plant material

Lyophilized leaf samples from 45 banana genotypes (36 allotriploid ABB and nine hybrid AB) were supplied by the Bioversity International *Musa* germplasm Transit Centre (ITC) hosted at KU Leuven, Belgium, except for three leaf samples collected in Indonesia during the Triangle collecting missions in 2012–13 (Hermanto *et al.*, 2014*a*, *b*) and originally considered as diploid AA accessions but revised as diploid AB (Christelová *et al.*, 2017), and currently conserved at the Indonesian Centre for Horticultural Research and Development (ICHORD). The passport data of these accessions are available in the Musa Germplasm Information System (MGIS) (Ruas *et al.*, 2017) (Table 1).

**Table 1.** List of 36 ABB and nine AB accessions used in this study. Cytotypes: Ca and Cb indicate chloroplasts originating from *M. acuminata* and *M. balbisiana*, respectively; Ma and Mb indicate the same for mitochondria

Acession code	Accession name	DOI	Genome*	Subgroup	Collection	Geographical origin	Cytotype	Genomic pattern
ITC0245	Safet Velchi	10.18730/9JM13	AB	Ney Poovan	ITC	India	CaMa	2*x*-1
ITC1034	Kunnan	10.18730/9M2KD	AB	Ney Poovan	ITC	India^†^	CaMa	2*x*-2
ITC1747	Agniswar	10.18730/9NGN7	AB	Kunnan	ITC	Indonesia	–	2*x*-2
ITC1729	Padali Moongil	10.18730/9NFE5	AB	Kunnan	ITC	India^†^	–	2*x*-3
ITC1752	Poovilla Chundan	10.18730/9NH1K	AB	Kunnan	ITC	India^†^	–	2*x*-3
ITC1751	Adukka Kunnan	10.18730/9NGYG	AB	Kunnan	ITC	India^†^	–	2*x*-4
Sum002	Muku Bugis	–	AB	–	ICHORD	Indonesia	–	(2*x*)
Sum004	Mu’u Seribu	–	AB	–	ICHORD	Indonesia	–	(2*x*)
ITC1880	Mu’u Pundi	10.18730/P5G84	AB		ITC	Indonesia (Sup008)	–	(2*x*)
ITC1700	Kepok Kuning	10.18730/9ND89	ABB	Saba	ITC	Indonesia	–	N/A
ITC1745	Kepok Tanjung	10.18730/9NGH3	ABB	Saba	ITC	Indonesia	–	N/A
ITC0632	Cachaco enano	10.18730/9KATX	ABB	Bluggoe	ITC	Colombia^‡^	–	1a
ITC0643	Cachaco	10.18730/9KBJG	ABB	Bluggoe	ITC	Colombia^‡^	–	1a
ITC1728	Sambrani Monthan	10.18730/9NFD4	ABB	Monthan	ITC	India	–	1a
ITC1746	Bankel	10.18730/9NGM6	ABB	Pisang Awak	ITC	India	–	1a
ITC1748	Boddida Bukkisa	10.18730/9NGQ9	ABB	Pisang Awak	ITC	India	–	1a
ITC1483	Monthan	10.18730/9MXQY	ABB	Monthan	ITC	India	CaMb	1a
ITC0026	Sabra	10.18730/9J6SQ	ABB	Unknown	ITC	Gabon	–	1a^+^
ITC0767	Dole	10.18730/9KGR$	ABB	Bluggoe	ITC	France^‡^	CaMb	1a^+^
ITC0361	Blue Java	10.18730/9JTR~	ABB	Ney Mannan	ITC	Fiji^‡^	–	1b
ITC1750	Ney Vannan	10.18730/9NGXF	ABB	Ney Mannan	ITC	India	–	1b
ITC1738	Kyauk Sein Phee Kyan	10.18730/9NG4V	ABB	Unknown	ITC	Myanmar	–	1b
ITC0123	Simili Radjah	10.18730/9JC16	ABB	Peyan	ITC	India	CbMb	1b^+^
ITC1600	INIVIT PB-2003	10.18730/9N676	ABB	Saba	ITC	Cuba^‡^	–	1c
ITC1138	Saba	10.18730/9M6MZ	ABB	Saba	ITC	Philippines	CaMb	1c
ITC0659	Namwa Khom	10.18730/9KCB4	ABB	Pisang Awak	ITC	Thailand	CbMb	2
ITC0053	Bom	10.18730/9J7YQ	ABB	Pisang Awak	ITC	Ivory Coast^‡^	–	2
ITC0087	Kayinja	10.18730/9J9YD	ABB	Pisang Awak	ITC	Burundi^‡^	–	2
ITC0101	Fougamou 1	10.18730/9JAX7	ABB	Pisang Awak	ITC	Gabon	–	2
ITC0526	Kluai Namwa Khom	10.18730/9K3PQ	ABB	Pisang Awak	ITC	Thailand^‡^	–	2
ITC1599	Kambani Zambia	10.18730/9N665	ABB	Pisang Awak	ITC	Tanzania	–	2
ITC1721	Karpuravalli	10.18730/9NEWR	ABB	Pisang Awak	ITC	India	–	2
ITC1737	Ya Khine	10.18730/9NG1R	ABB	Unknown	ITC	Myanmar	–	2
ITC0339	Pisang Awak	10.18730/9JSGY	ABB	Pisang Awak	ITC	Australia^‡^	–	2^+^
ITC1719	Chinia	10.18730/9NERM	ABB	Pisang Awak	ITC	India	–	3
ITC1749	Vananthpurani	10.18730/9NGTC	ABB	Pisang Awak	ITC	India	–	3
ITC0472	Pelipita	10.18730/9K0MU	ABB	Unknown	ITC	Philippines	CaMb	4
ITC0396	Pelipita	10.18730/9JW32	ABB	Unknown	ITC	Costa Rica^‡^	–	4
ITC0397	Pelipita Majoncho	10.18730/9JW65	ABB	Unknown	ITC	Costa Rica^‡^	–	4
ITC0652	Kluai Tiparot	10.18730/9KC0Y	ABB	Unknown	ITC	Thailand	CbMb	5
ITC0473	Balonkawe	10.18730/9K0P1	ABB	Klue Teparod	ITC	Philippines	–	5
ITC0983	Auko	10.18730/9KZKW	ABB (M)	Unknown	ITC	PNG	–	6
ITC0987	Auko	10.18730/9KZZ3	ABB (M)	Unknown	ITC	PNG	–	6
ITC0990	Vunapope	10.18730/9M06A	ABB (M)	Unknown	ITC	PNG	–	6
ITC1682	Chuoi mit	10.18730/9NC06	ABB (AB)	Unknown	ITC	Vietnam	–	7

*(AB), genome erroneously classed or labelled as AB in taxonomic classification (passport data) but recognized as triploid in Christelova *et al.* (2017); (M), erroneously classed as AB in taxonomic classification and reported as mixoploidy (2*x* + 3*x*).

^†^Assumed geographical origin from literature or name.

^‡^Geographical origin of the previous *ex situ* collection.

^+^Genomic pattern containing aneuploidy regions.

### DNA extraction and RADSeq data generation

Genomic DNA was extracted using the 2X CTAB protocol. The method used to create the library for restriction-site-associated DNA sequencing (RADSeq) used the PstI restriction enzyme. The 300–500 short-insert libraries were sequenced with 91 bp paired-end reads using Illumina HiSeq2000 (Illumina, San Diego, CA, USA) by BGI Hong Kong. At BGI, the raw data were modified with the following two steps: (1) reads polluted by adapter sequences were deleted; and (2) reads that contained >50 % low-quality bases (quality value ≤5) or >10 % N bases were removed.

### Read mapping, filtering and SNP calling

Paired-end reads contained in raw FASTQ files were checked using FastQC. Reads were then cleaned to remove adapter sequences and low-quality ends (Phred score >20) with Cutadapt (Martin, 2011). After trimming, reads inferior to 30 bp were discarded. Reads were then aligned using BWA (Li and Durbin, 2009) with the default parameter against the *Musa acuminata* genome of the reference v2 (DH Pahang) (D’Hont *et al.*, 2012; Martin *et al.*, 2016) downloaded from the Banana Genome Hub (Droc *et al.*, 2013). Read groups were added for each alignment and reads were locally realigned with IndelRealigner (Genome Analysis ToolKit) (McKenna *et al.*, 2010). HaplotypeCaller from GATK version 3.4-46 was then used to get a gVCF file for each accession. Genotyping on gVCF files was performed with GenotypeGVCFs from GATK 3.4-46. SNPs were called on uniquely mapped reads with HaplotyCaller from GATK version 3.4-46.

### Detection of homologous exchange

The subgenomic structure was investigated using VcfHunter (https://github.com/SouthGreenPlatform/VcfHunter). Based on known sequence variability in the A and B genomes, SNP variants were assigned to the ancestral genomes in order to plot the A/B genome allele coverage ratio and to calculate normalized site coverage along chromosomes as described in Baurens *et al.* (2019). SNP datasets from accessions representing *M. acuminata* and *M. balbisiana* sequence variability were retrieved with GIGWA (Sempéré *et al.*, 2016) on MGIS (https://www.crop-diversity.org/mgis/gigwa) (Ruas *et al.*, 2017). Homologous exchanges (HEs) were inferred when, in a given chromosome, a change in A/B allele ratio was observed between adjacent regions. Chromosomes were identified by their number in the A genome reference (from 01 to 11) (D’Hont *et al.*, 2012) preceded by ‘chr’. Chromosome arms were defined as ‘first’ or ‘second’ according to the chromosome sequence.

### Diversity analysis and accession relationship

The SNPRelate package in R (v1.18) (Zheng *et al.*, 2012) was used to investigate genetic diversity of triploid bananas. The Vcf file obtained was converted into the genomic data structure (GDS) file format. Only bi-allelic SNPs were selected to build the dendrogram. A dissimilarity matrix between samples was built with SNPRelate with a hierarchical clustering R package (Suzuki *et al.*, 2006; pvclust). The statistical support of the genetic relationship among accessions was determined by running 100 bootstrap replicates. The resulting tree in Newick format was produced with MEGA6.

## RESULTS

### Molecular karyotyping

A set of nine AB and 36 ABB genotypes were used to identify SNP markers using the RADSeq method. The number of SNPs ranged between 10 000 and 26 000 per chromosome for each genotype (182 000 SNPs by genotype on average) (Supplementary Data Material 1).

As in Baurens *et al.* (2019), SNPs were assigned to the A and B subgenomes and used to scan chromosomes for the detection of deviating regions, i.e. portions of chromosomes where the subgenome ratios differ from the expected ratios based on the assumed genomic composition (i.e. A1:B1 and A1:B2 for AB hybrids and ABB allotriploids, respectively), also referred to as HEs.

### AB hybrids

**Fig. 1.**
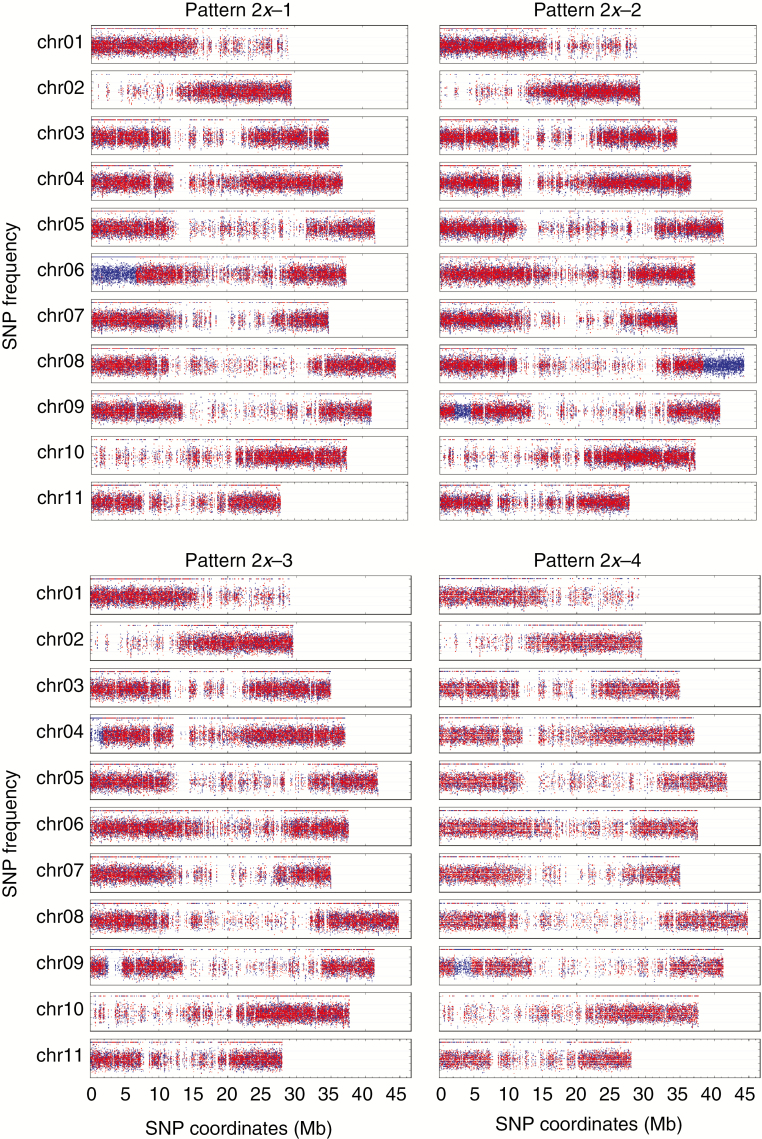
Patterns of AB hybrids having genomic regions with an unexpected subgenome ratio. Frequencies of SNPs (*y-*axis) assigned to A and B genomes are shown in blue and red, respectively. SNP coordinates (*x*-axis) are reported in Mb at the bottom of each genotype figure.

A panel of nine banana accessions reported as AB hybrids was analysed (Table 1). Only one-third of them (three) had the expected subgenome composition (i.e. A1:B1), while the others (six) showed one or two deviating regions with an A2:B0 ratio (i.e. missing B-specific SNPs). By comparing all HEs together we delineated four patterns, denoted as 2*x*-1, 2*x*-2, 2*x*-3 and 2*x*-4. The cultivar ‘Safet Velchi’ had a A2:B0 ratio in the first-arm terminal region of chr06 (Fig. 1, pattern 2*x*-1); cultivars ‘Kunnan’ and ‘Agniswar’ showed an A2:B0 ratio in the terminal region of second arm of chr08 and in a first-arm interstitial region of chr09 (Fig. 1, pattern 2*x*-2). ‘Padali Moongil’ and ‘Poovilla Chundan’ showed an A2:B0 ratio in the first-arm terminal region of chr04 and in a first-arm interstitial region of chr09 (Fig. 1, pattern 2*x*-3); ‘Adukka Kunnan’ had an interstitial A2:B0 region in the first arm of chr09 (Fig. 1, pattern 2*x*-4). Among these samples, the cytotypes of ‘Safet Velchi’ and ‘Kunnan’ were previously characterized with chloroplasts and mitochondria originating from *M. acuminata* (Carreel *et al.*, 2002; Boonruangrod *et al.*, 2008).

### ABB allotriploids

**Fig. 2.**
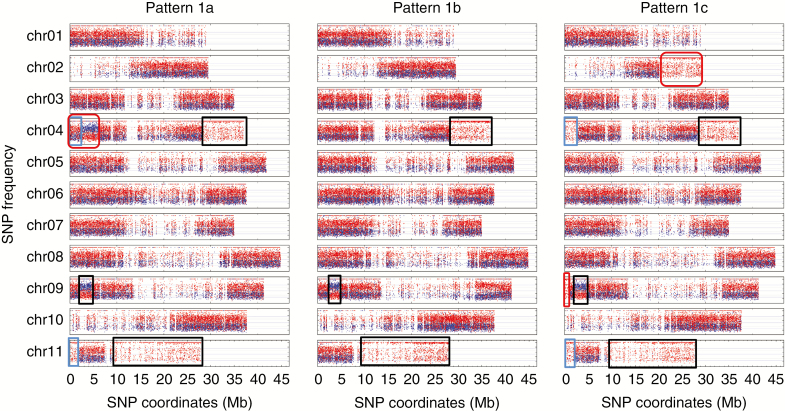
Patterns 1a, 1b and 1c of ABB allotriploids having genomic regions with an unexpected allele ratio composition. Frequencies of SNP (*y*-axis) assigned to A and B genomes are shown in blue and red, respectively. SNP coordinates (*x*-axis) are reported in Mb at the bottom of each genotype figure. Black rectangles indicate regions shared by all three patterns, blue rectangles indicate regions shared by patterns 1a and 1c, and red boxes indicate regions specific to the respective pattern.

**Fig. 3.**
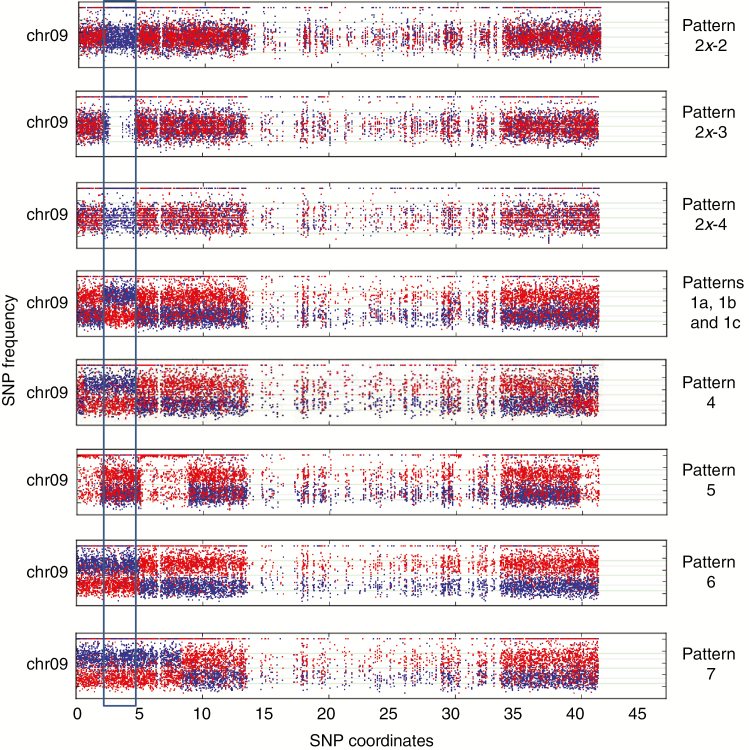
Chromosome 09 pseudo-karyotypes observed in three AB and seven ABB patterns. The rectangle highlights the A-genome-enriched region in all chromosomes 9. Frequency of SNPs (*y*-axis) assigned to A and B genomes are shown in blue and red, respectively.

In 34 out of the 36 ABB genotypes, HEs with a subgenome ratio corresponding to A0:B3 (region with only B-assigned SNPs) or A2:B1 (region with two-thirds A-, one-third B-assigned SNPs) were detected. None with an A3:B0 ratio were found. Nine patterns corresponding to specific combinations were identified, enabling the classification of each genotype (Table 1). Three patterns sharing regions with the same ratio deviation and size were numbered 1a, 1b and 1c. In these, large terminal regions of the second arm of chr04 and chr11 had an A0:B3 ratio and the first arm of chr09 showed an A2:B1 interstitial region (Fig. 2). Patterns 1a and 1c had in common the A0:B3 terminal region of the chr11 first arm (Fig. 2) but pattern 1a differed, with a specific interstitial region A2:B1 in chr04 first arm. Pattern 1c had three specific regions A0:B3, in chr02 second arm, chr04 first arm and in chr09 first arm. The interstitial region in chr09 where the A genome replaced the B counterpart had a size very similar or identical to that shared by the 2*x*-2, 2*x*-3 and 2*x*-4 patterns in AB hybrids (Fig. 3). These patterns contain mostly genotypes belonging to the Bluggoe, Monthan, Saba, Peyan and Ney Mannan subgroup, which originated from India.

**Fig. 4.**
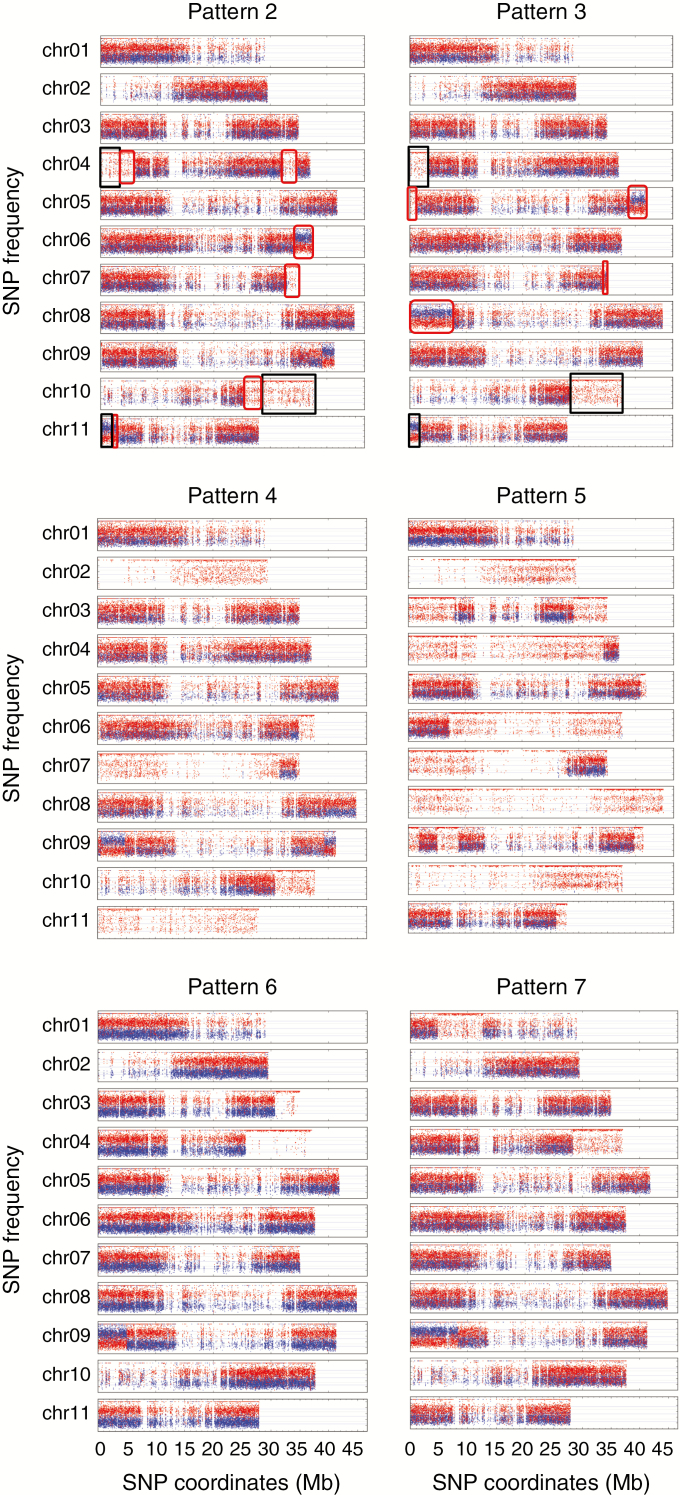
Patterns 2–7 of ABB allotriploids having genomic regions with an unexpected allele ratio composition. Frequencies of SNP (*y*-axis) assigned to A and B genomes are shown in blue and red, respectively. SNP coordinates (*x*-axis) are reported in Mb at the bottom of each genotype figure. Black rectangles indicate recombined regions partially overlapping in the patterns 2 and 3; red boxes indicate recombined regions differentiating patterns 2 and 3.

The remaining patterns did not share identical HEs and were named from 2 to 7. Patterns 2 and 3 grouped genotypes classified as Pisang Awak (Fig. 4), with the exception of two assigned to pattern 1a that are known to be erroneously allocated to this subgroup (Christelová *et al.*, 2017). Patterns 4 and 5 were the only ones having entire chromosome substitutions with the presence of an A0:B3 subgenome ratio. Specific to pattern 5, no regions with an A2:B1 ratio were detected. Overall, patterns 4 and 5 had an excess of B subgenomes compared with other ones (Fig. 4) and corresponded to genotypes assigned to subgroups Pelipita and Klue Teparod, respectively. Pattern 6 was not assigned to any known subgroup but the three genotypes composing it shared the same origin from Papua New Guinea (PNG) (Fig. 4, Table 1). Pattern 7 was represented by only one genotype, ‘Chuoi mit’, from Vietnam (Fig. 4). Finally, two genotypes (‘Kepok Kuning’ and ‘Kepok Tanjung’, originally classified as belonging to subgroup Saba) both originating from Indonesia, did not show any region with a deviating subgenome ratio and were not associated with any pattern.

### Genotype-specific aneuploidies

**Fig. 5.**
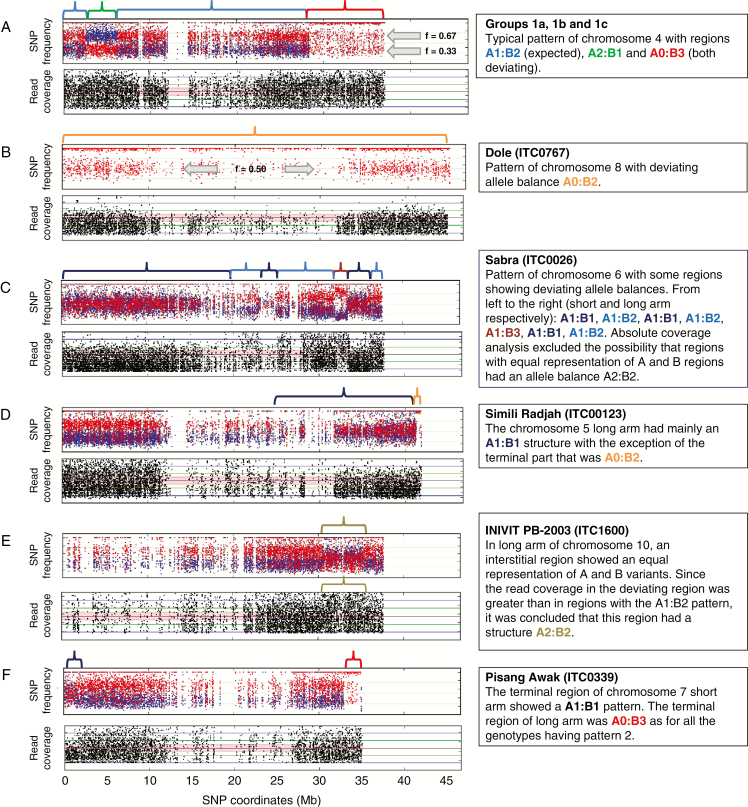
Patterns of critical chromosomes involved in aneuploidy. For each pattern, frequencies of SNPs assigned to A and B subgenomes (shown in green and red, respectively) and absolute read coverage (shown in black in the lower graphic in each panel) are reported for chromosomes with a deviating pattern. (A) Pattern of chromosomes present in the expected number of copies (three). (B–F) Patterns with changes in number of copies involving entire or partial chromosomes. Curly bracket colours indicate regions with the inferred subgenome composition. Coloured curly brackets delimitate the regions with inferred genome ratios reported in the box on the right. Grey arrows illustrate different allele frequencies (*f*) used to discriminate between regions with A0:B3 and A0:B2 ratios in a and b patterns.

For five genotypes, in addition to the described HEs based on ratios involving three chromosomes (Fig. 5A), portions or entire chromosomes displayed ratios with two or four copies. For the ‘Dole’ genotype belonging to the Bluggoe subgroup (pattern 1a), no SNPs assigned to the A subgenome were detected along the whole of chr08. The lower SNP coverage compared with other chromosomes and the unimodal coverage distribution of residual B genome heterozygosity (at 0.5) indicates an A0:B2 ratio, implying the A version of chr08 was lost (Fig. 5B). The chr06 of ‘Sabra’ (pattern 1a) shows a complex structure (Fig. 5C). On the first arm there is similar coverage of A- and B-assigned SNPs around 0.5 and lower SNP coverage than other regions, compatible with an A1:B1 subgenome ratio. On the second arm, six regions with A1:B1, A1:B2 and A1:B3 ratios were inferred. In ‘Simili Radjah’, a large part of the second arm of chr05 is missing. Most of the region exhibits a diploid pattern A1:B1 except in its terminal region, where A0:B2 appears (Fig. 5D). The genotype ‘INIVIT PB-2003’ (pattern 1c) showed a unique interstitial region, in the chr10 second arm, where the SNPs assigned to A and B had similar contributions. Since SNP coverage in this region was higher than in the rest of chr10, an A2:B2 homoallele ratio was inferred (Fig. 5E). Finally, the accession ‘Pisang Awak’ (ITC0339, pattern 2) had an A1:B1 homoallele ratio in the terminal part of the chr07 first arm (Fig. 5F).

Since these aneuploidies were specific to single genotypes (not shared with other genotypes with the same HE pattern), we considered that mutation events occurred after the original triploidization events.

### Diversity analysis

We used SNPs from genomic regions not involved in any HE in 31 ABB genotypes (pattern 4 and 5 genotypes, with high B genome content, were excluded from the analysis) to perform a genetic diversity analysis. In the diversity tree, two very well supported clusters can be distinguished (Supplementary Data Material 2). The first cluster includes the 11 accessions having patterns 2 and 3, whereas the second comprises all the remaining genotypes.

## DISCUSSION

In this study, genome-wide molecular markers (RADSeq-derived SNPs) were used to characterize the genome of diploid and triploid banana hybrid cultivars (with an emphasis on the ABB group) with the objective of implementing an efficient classification system for germplasm management. RADSeq is an affordable method that provides high coverage of markers along the chromosomes. By applying the software proposed in Baurens *et al.*, 2019, we unravelled the HEs in banana accession samples as specific signatures of the taxonomic subgroups. Since HE detection is based on allele ratio changes, reciprocal HEs possibly present in the analysed genomes could not be detected.

Such genome structural rearrangement occurs frequently in polyploid crops, such as rapeseed, cotton, mustard rape, strawberry and bread wheat (Salmon *et al.*, 2010; Guo *et al.*, 2014; He *et al.*, 2017; Lloyd *et al.*, 2018; Edger *et al.*, 2019), and can have consequences for gene expression (Lloyd *et al.*, 2018; Cenci *et al.*, 2019; Zhang *et al.*, 2019) and the presence/absence or copy number variations of genes linked to agronomic traits (Stein *et al.*, 2017; Hurgobin *et al.*, 2018). For banana, we show that HEs are useful in gaining new insights into the evolutionary events that led to the creation of allopolyploids.

### The complex history of ABB cultivars can be grouped into at least nine founding events

The classification of cultivated ABB bananas has long needed clarification. (De Langhe 2002) noted that ‘The more popular names (Saba, Pisang Awak, Peyan, Bluggoe, Monthan) actually represent a cluster of closely related cultivars, generated by somatic variation’. Each variant has its local name in Asia, which makes the nomenclature of the whole ABB group difficult to resolve based solely on this source of information. The combination of morpho-taxonomic descriptors and SSR markers confirmed this difficulty in discriminating the Indian subgroups (Saraswathi *et al.*, 2011), and the use of diversity arrays technology (DArT) or SSR markers on a wider sample showed that the classification was consistent for accessions belonging to the subgroups Pelipita, Klue Teparod and Pisang Awak (Sardos *et al.*, 2016; Christelová *et al.*, 2017). However, accessions classified as belonging to the subgroups Saba, Monthan, Bluggoe, Ney Mannan or Peyan were often misclassified (Sardos *et al.*, 2016).

**Fig. 6.**
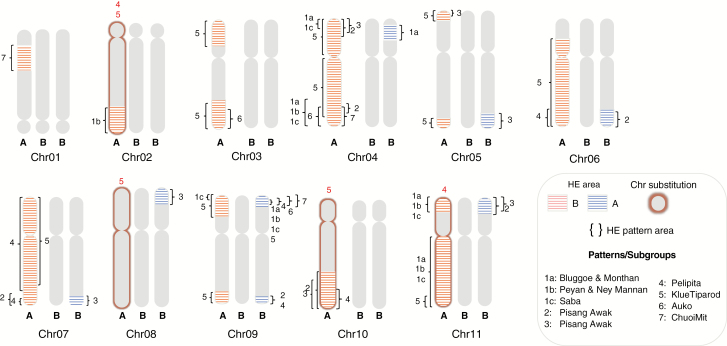
Schematic ideogram overview of all HEs and chromosome substitutions detected in the 36 ABB bananas. Hatching patterns indicate the approximate area (cumulated when overlapping) of HEs between A (blue) and B (red). Numbered brackets indicate the boundaries for a specific pattern number. Chromosome substitutions are illustrated with the chromosome border highlighted in colour (red for B).

Overall, all the allotriploid accessions were confirmed as having an ABB genome constitution, i.e. the ratio between variants assigned to B and A subgenomes was A1:B2. We further explored the presence of HEs in a comprehensive set of ABB cultivars, as initiated in previous studies (Baurens *et al.*, 2019; Cenci *et al.*, 2019; Wang *et al.*, 2019), and confirmed that such a phenomenon has been very common in the creation of these domesticated bananas. Among the 36 genotypes surveyed, 34 had at least two chromosome portions for which the observed ratio was either A2:B1 or A0:B3 (Supplementary Data Material 3; Fig. 6).

In total, 53 HEs were inferred in the nine ABB and four AB patterns (shared HEs were counted only once) (Supplementary Data Material 3). They were found in all 11 *Musa* chromosomes but were unevenly distributed, with 11 and 13 independent recombination events in the most recombined chr04 and chr09, respectively, and only one recombination event in chr02 (Supplementary Data Material 3; Fig. 6). Since no recombination hotspots were observed for chr04 and chr09 by Baurens *et al.* (2019) using segregating populations, the high frequency of HEs we detected in these chromosomes may be due to the presence of genes influencing domestication traits for which human selection could have favoured some combinations between A and B homoalleles. In particular, the first arm of chr09 appears to be involved in several independent HEs, and a subgenome A interstitial region was present in all the cultivars analysed (Fig. 3). Indeed, three AB hybrids and patterns 1a, 1b and 1c had identical or very similar HE coordinates but interstitial or terminal regions on patterns 4, 5, 6 and 7 had different HE coordinates, implying different events. This supports the hypothesis that the presence of this A homoallele was selected during the domestication process.

The different HE patterns of ABB allotriploidy provide new insights into their current classification, as discussed below.

### West ABB

#### Bluggoe, Monthan, Peyan and Ney Mannan share the same common origin.

The dominance of accessions from India in patterns 1a and 1b is obvious. Equally, diploid hybrids (AB) from India appear to share HEs with patterns 1. We can therefore locate the early origin of the recombination events typical of patterns 1 in India. All cultivars of subgroup Bluggoe (Table 1), but also Monthan, showed HE pattern 1a. Subgroups Peyan and Ney Mannan both had pattern 1b. This study shows that Bluggoe, Monthan, Peyan and Ney Mannan share a common origin linked to India. Two additional Indian cultivars, classified as Pisang Awak, shared pattern 3.

### East ABB

#### Pisang Awak subgroup would originate from at least two independent events

Eight Pisang Awak cultivars were included in pattern 2 (Fig. 4). Comparing patterns 2 and 3 (i.e. coordinates of break points), we noticed that despite the independent occurrence of the recombination events in each pattern, similar deviations in A and B ratios were present in three chromosomal regions (black squares in Fig. 4). Such results could suggest that these regions harbour genes giving similar phenotypic traits to the cultivars with patterns 2 and 3 and that these traits influenced their classification in the same subgroup. However, phenotypic proximity among the cultivars of these two HE patterns likely results from genetic similarity in parental genotypes (Christelová *et al.*, 2017). The diversity tree based on SNPs in genomic regions with ABB composition indicated that genotypes having patterns 2 and 3 form a group clearly differentiated from the other accessions. Since regions involved in HE were excluded from the analysis, no bias due to overlapping regions with similar HEs is expected in these results. The diversity analysis confirmed that the parents contributing to both Pisang Awak patterns had a high level of genetic similarity. Consequently, it remains difficult to conclude that the phenotypic similarity between those genotypes is due to their global genetic background or to the similarity in recombination patterns.

### Pelipita

All accessions (three) classified in the Pelipita subgroup shared an identical and specific deviating pattern (pattern 4; Fig. 4). These cultivars [‘Pelipita’ (ITC0396 and ITC0472) and ‘Pelipita Majoncho’] had an entire A chr02 and chr11 substitution (A0:B3 structure) and chr07 was mainly A0:B3, with the exception of the second-arm interstitial region. These findings in the ‘Pelipita’ genome are consistent with observations made through *in situ* hybridization reporting that ‘Pelipita’ contained 25 B and eight A chromosomes (D’Hont *et al.*, 2000). Additional chromosome regions with an unexpected ratio were also present, notably two regions of chr09 exhibiting ratios favouring A (A2:B1).

### Klue Teparod

The two cultivars of HE pattern 5 (‘Kluai Tiparot’ and ‘Balonkawe’) could be easily distinguished from all the other surveyed accessions. First, all deviating regions were A0:B3 (no A2:B1 were observed, contrary to all other patterns); and second, three entire chromosomes (chr02, chr08 and chr10) were fully B and three additional A0:B3 centromeres were present in recombined chromosomes (Fig. 4). This observation provides new insights into a long-lasting debate that exists on the classification of the Klue Teparod subgroup. The ‘Balonkawe’ accession, originally collected in the Philippines by Allen in 1959, was first classified as a tetraploid ABBB due to its robust appearance (Rosales *et al.*, 1999). However, flow cytometry coupled with chromosome counting indicated that the ‘Klue Tiparot’ accession from the same subgroup was in fact a triploid (Jenny *et al.*, 1997). Later, the use of several types of molecular marker led to a different conclusion. Ribosomal DNA (Boonruangrod *et al.*, 2008) suggested the presence of B genomes only while internal transcribed spacer (ITS) sequences confirmed the occurrence of A (Hřibová *et al.*, 2011; Čížková *et al.*, 2013). Dominant DArT markers that resulted in the clustering of these two accessions into the *M. balbisiana* gene pool led to the hypothesis of the presence of an incomplete A genome (Sardos *et al.*, 2016). In this study, it is shown that Klue Teparod accessions contain a small fraction of an A genome, which makes this subgroup the cultivated variety carrying the highest content of B genome ever described. Similar to the cultivar ‘Lep Chang Kut’ (ITC0647), registered as BBB in MGIS (Ruas *et al.*, 2017) but for which the presence of A isoforms was detected (Carpentier *et al.*, 2011), we provide evidence that Klue Teparod’s accessions are not parthenocarpic BBBs.

### Saba

The four cultivars classified as Saba in our sample showed two different patterns: two Indonesian cultivars showed a strict A1:B2 ratio whereas the other two of unknown origin showed the 1c pattern. Although a group of cooking cultivars from the Philippines, including ‘Saba’ (not in our samples), were reported to be BBB (Valmayor *et al.*, 1991; Sales *et al.*, 2010), no such pattern was identified in the present study. However, it is confirmed that the same vernacular name is used for genotypes with different ancestral origins. Pattern 1c shared some HEs with Indian patterns 1a and 1b, suggesting that these cultivars were also derived from an ancestor originating in the Indian subcontinent.

### Undescribed subgroup

The genotype ‘Chuoi mit’ (ITC1682) [misclassified as AB but determined as triploid by Christelová *et al.* (2017)] exhibited its own specific HE pattern (pattern 7) (Fig. 4). Morphological and genomic studies of additional genotypes would be required to support the creation of a new subgroup.

### West Oceania ABB

In this study, ABB accessions from PNG revealed a specific pattern (pattern 6). Given the absence in this analysis of genotypes belonging to the Kalapua subgroup, originating from PNG, we could not investigate its HE pattern. Therefore, it should be investigated whether Kalapua accessions share the same HE pattern 6 (Fig. 4) or whether an additional pattern from PNG exists. This would be a rationale to distinguish East ABB and West Oceania, which are usually combined but originated from different continents separated by the Wallace Line.

### Genesis of HE patterns

The discovery of nine founding events opens the discussion on the possible routes that led to the creation of these different subgroups and the formulation of new hypotheses. To obtain a triploid genome constitution (2*n* = 3*x*), a diploid gamete (*n* = 2*x*) needs to meet a haploid gamete of the opposite sex (*n* = *x*). Usually, 2*x* gametes can be produced by tetraploid genotypes (2*n* = 4*x*) or by a diploid genotype (2*n* = 2*x*) through a meiosis failing one of the divisions [i.e. first- or second-division restitution (FDR or SDR, respectively)] and ending with a doubled chromosome set. Hybrids from different species are prone to setting unreduced gametes, derived from FDR (Ramsey and Schemske, 1998).

In banana it has also been reported that allotriploids can produce viable reduced gametes, both haploid (*n* = *x* = 11) and diploid (*n* = 2*x* = 22) (Shepherd, 1999).

**Fig. 7.**
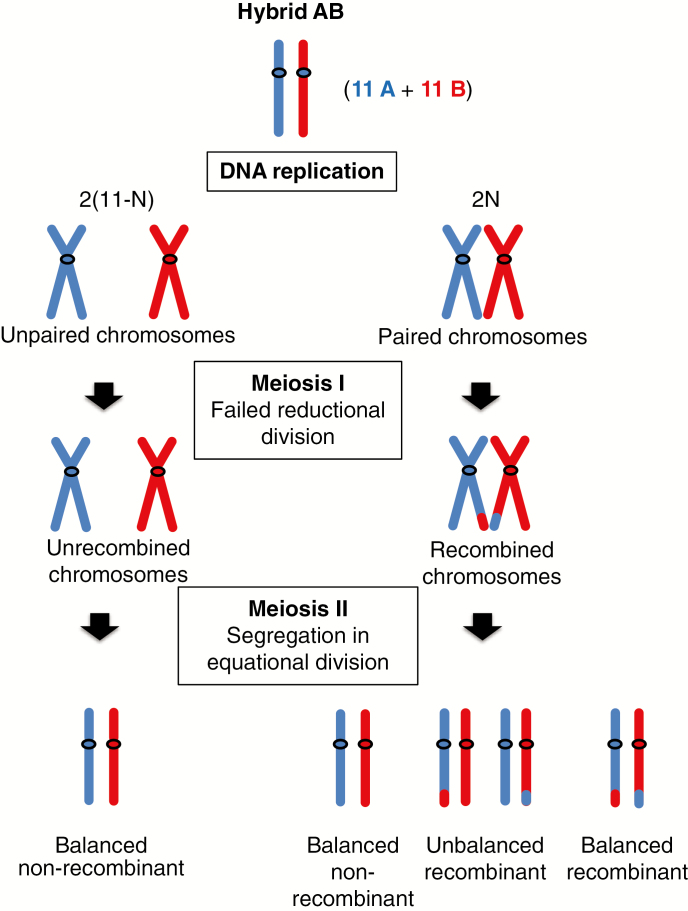
Scheme of chromosome fates in a hybrid AB meiosis producing unreduced gametes. Blue and red colours represent chromosomes from *M. acuminata* (A genome) and *M. balbisiana* (B genome), respectively. Recombinations are possible only between homologous chromosomes that have undergone pairing. Recombinant chromosomes are detectable by SNP allele analysis only if genome portions are unbalanced after segregation in the second meiotic division (equational).

To obtain an ABB constitution, two gametic combinations (GCs) are consequently possible:

- AB × B: a 2*x* gamete containing A and B genomes originating from (1) a hybrid genotype (AB) by FDR, or (2) an allotriploid (AAB or ABB) or (3) an allotetraploid (AABB) that met a normal *M. balbisiana* gamete (B). In the first case the unreduced AB gamete brings 11 A and 11 B centromeres with possible HEs due to partial pairing between homologous chromosomes (Fig. 7). In the second case HEs are also possible but an unbalanced A/B centromere representation is expected due to the double dose of one of the two subgenomes present in the allotriploid genotype. In the third case, 11 A and 11 B chromosomes are expected due to the regular meiosis between homologous chromosomes, but consequently homologous pairing producing HEs is expected to be infrequent.
- A × BB: a haploid *M. acuminata* gamete (A) meets a gamete (BB) originated from *M. balbisiana* by unreduced meiosis. Here, recombination between A and B genome cannot take place before the establishment of allotriploidy.

For the two genotypes from Indonesia without detected HEs, both GCs are possible and cannot be discriminated by our method due to the possible presence of invisible balanced HEs (Fig. 7). Such case of balanced HE was probably revealed in the second arm of chr05 because of the aneuploidy in ‘Simili Radjah’ genotype (Fig. 5D) as none of the accessions with the same HE pattern (1b) showed a deviating ratio for this chromosome (Supplementary Data Material 4).

### Routes involving primary AB hybrids

Based on our observations of unbalanced regions between A and B genomes in allotriploids, we suggest that the diploid gamete contributing to the allotriploid with patterns 2, 3, 6 and 7 originated from a nucleus restitution during a diploid hybrid (AB) meiosis, which generated the observed chromosome recombinations (Fig. 7). De Langhe *et al.* (2010) hypothesized that this route would be restricted to India due to the exclusive occurrence of AB in this country. However, since then AB hybrids have also been collected in Indonesia (Hermanto *et al.*, 2014*a*, *b*), making possible routes involving AB hybrids also for ABB genotypes with origins in South-East Asia and New Guinea.

### Routes leading to shared HEs

The observed sharing of some unbalanced regions (Fig. 2) in HE patterns 1a, 1b and 1c cannot be explained by a simple AB × B gamete combination. In fact, identical or very similar deviating patterns (three unbalanced regions corresponding to these three recombination events) are unlikely to be obtained three times by fully independent meiosis, and must have been transmitted independently through common ancestry. In other words, the occurrence of partially shared patterns suggests repeated inheritance of the same HEs. Indeed, the reported differences in chloroplast origin (Carreel *et al.*, 2002; Boonruangrod *et al.*, 2008) support independent crossing pathways for patterns 1a, 1b and 1c (Table 1).

To explain this observation, we hypothesize that the shared HEs were already present in AB hybrids, which inherited them from A and B gametes previously introgressed. These hybrids then produced unreduced gametes in which new HEs possibly occurred during meiosis and gave rise to the allotriploid ancestors of the three patterns 1a, 1b and 1c. Some recombination events could also have even been provided by *M. balbisiana* haploid gametes contributing the second B subgenome. This route could be formulated as (A^b^B^a^) × B^a^B^a^ → A^b^B^a^B^a^. A possible scenario leading to patterns 1a, 1b and 1c is proposed in Supplementary Data Material 5.

**Fig. 8.**
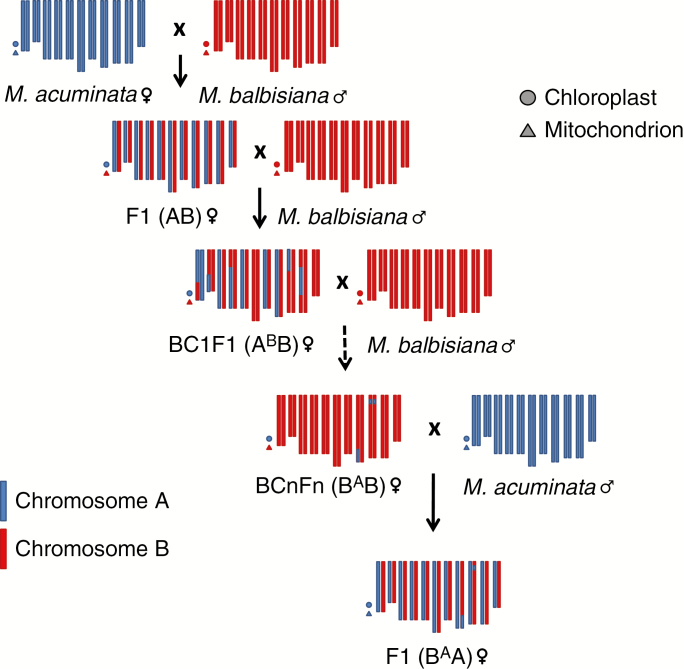
Scheme of backcrosses generating introgressions of A genome in the *M. balbisiana* parent of AB hybrids having genomic regions with an unexpected subgenome ratio. Blue and red colours represent chromosomes from *M. acuminata* (A genome) and *M. balbisiana* (B genome), respectively.

This hypothesis is supported by the observation of hybrid AB genotypes containing unbalanced regions (Fig. 1) in which four different HE patterns were found. These results imply that the respective *M. balbisiana* donor plants were all introgressed with A genome portions. This suggests genetic fluxes between *M. acuminata* (or AA) and *M. balbisiana*, particularly in India, where these unbalanced AB hybrids and ABB allotriploids originated and where cultivated *M. balbisiana* are abundant (Subbaraya *et al.*, 2006). Since in *Musa* chloroplasts and mitochondria are inherited from the female and male gametes, respectively (Fauré *et al.*, 1993) and both hybrid AB cytotypes have an A origin for both chloroplasts and mitochondria, the probable crossing scheme leading to the creation of these hybrids must have involved *M. acuminata* or AA gametes at least twice (Fig. 8). However, this implies the incomplete sterility of AB hybrids, which are able to produce not only unreduced gametes (*n* = 2*x* = 22) but also viable reduced gametes (*n* = *x* = 11), where A and B chromosomes recombine as in an intraspecific context and segregate.

Interestingly, in these genotypes an interstitial region in the first arm of chr09 exhibits very similar HEs in allotriploids and diploids, which again suggests common ancestry (Fig. 3). As already observed by Baurens *et al.* (2019), the subtelomeric region of the first arm of chr09 often appears to be enriched in the A genome (only patterns 2, 3 and 2*x*-1 are not). In our sample, in addition to the above-mentioned diploid and triploid HE patterns, four patterns have independent HEs where the A genome has replaced the B counterpart in the subtelomeric region of the first arm of chr09 (Fig. 3).

### Routes leading to a B excess ratio

Patterns 4 and 5 are both characterized by a higher presence of the B genome, with three and six B centromeres having replaced the A counterpart, respectively. The absence of A centromeres (Fig. 4) is not consistent with a meiosis of an AB hybrid where FDR gametes were produced by a failed first meiotic division. On the other hand, 2*x* gametes produced by the failure of equational division (SDR) are not expected to have an excess of the B genome or to lack centromeres with an A2:B1 constitution, as observed in patterns 4 and 5.

**Fig. 9.**
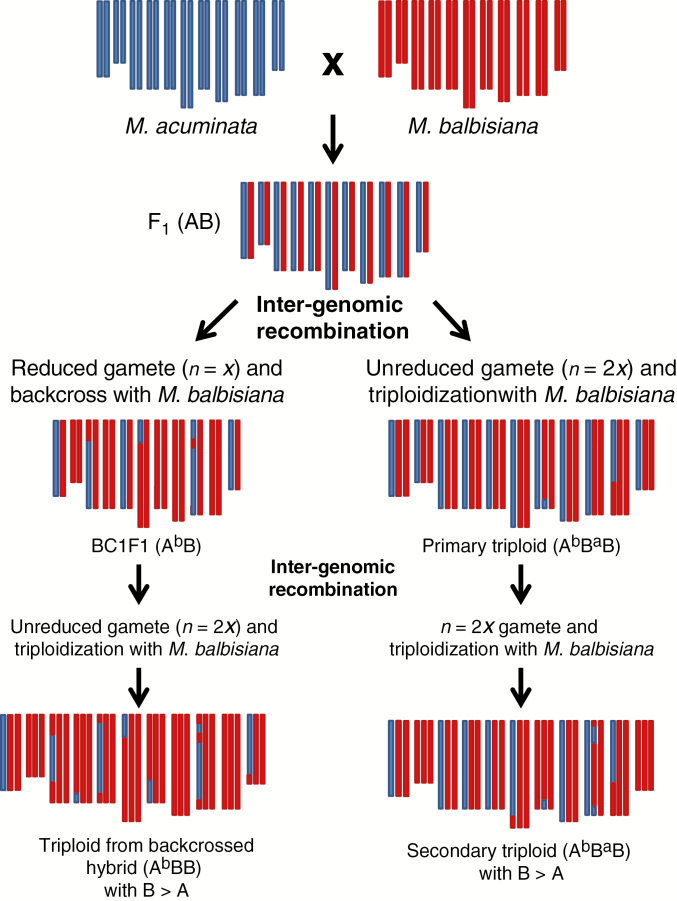
Scheme of possible crossing pathways generating B-genome-rich ABB patterns 4 and 5. Blue and red colours represent chromosomes from *M. acuminata* (A genome) and *M. balbisiana* (B genome), respectively.

The larger contribution of *M. balbisiana* to these cultivars is consistent with an unreduced *n* = 2*x* gamete originating from a backcrossed diploid hybrid (originating in an *n* = *x* gamete from an AB hybrid that met an *n* = *x M. balbisiana* gamete) crossed with a regular B gamete (Fig. 9). An unreduced gamete from such a backcrossed hybrid (A^b^B or B^a^B) is expected to have pairs of B centromeres for approximately half of its chromosomes and A/B heterozygous centromeres for the other half (Fig. 9). This can be formulated as AB × BB → A^b^B (or B^a^B) × BB → A^b^BB (or B^a^BB).

Alternatively, it can be hypothesized that a B-enriched *n* = 2*x* gamete could be produced by an ABB allotriploid. In this case, according to random centromere segregation, one-third of the centromeres are expected to be homozygous for B and two-thirds are expected to be A/B heterozygous (Fig. 9). This would be formulated as ABB × BB → A^b^BB.

Pattern 4, with three of 11 centromeres having an A0:B3 ratio, fits with the second hypothesis, whereas pattern 5, with six of 11 centromeres A0:B3, is more in agreement with first one. However, due to the low number of *Musa* chromosomes (11), those hypotheses cannot be excluded for both patterns. Somehow, independent origin of patterns 4 and 5 is supported by the completely different HEs and by the different crossing history that is inferred from the organelle origin. ‘Kluai Tiparot’ (pattern 5) has both organelles originating from *M. balbisiana*, whereas ‘Pelipita’ (pattern 4) has chloroplasts inherited from *M. acuminata* and mitochondria from *M. balbisiana* (Boonruangrod *et al.*, 2008).

### Conclusions and perspectives

The results of this study demonstrate multiple origins of ABB cultivars, with different and sometimes complex routes. It is possible that extension of RADSeq genome surveying to other cultivars will allow the discovery of additional HE patterns, in both AB hybrids and in allotriploids. The frequent occurrence of HEs indicate that A and B subgenomes are prone to recombination, making the *M. balbisiana* genome a source of potential useful variability to create new cultivars able to answer the numerous challenges in banana breeding. As an example, ABB bananas are hypothesized to be more drought-tolerant (van Wesemael *et al.*, 2019). Knowing the origin of our current cultivars, and thus their potential parents, will help breeders make the right choices for future crosses.

Compared with previous analysis methods to assess the genetic diversity of the banana gene pool, whole-genome surveying based on next-generation sequencing (NGS; RADSeq in this study) provides a detailed and exhaustive picture of the genome composition. Therefore, we recommend complementing SSR-based characterization with NGS technologies such as RADSeq as a standard method to characterize banana gene bank accessions and to classify them in an objective, timely and repeatable way.

## SUPPLEMENTARY DATA

Supplementary data are available online at https://academic.oup.com/aob and consist of the following. Material 1: number of SNPs assigned to each chromosome and to chloroplasts/mitochondria in the analysed cultivars. Material 2: diversity tree based on SNPs in regions not involved in HE in any of the analysed genotypes. Material 3: number of HEs in ABB and AB patterns. Material 4: hidden HEs revealed by aneuploidy. Chr05 pattern of ‘Blue Java’ and ‘Simili Radjah’. Material 5: schema of possible crossing pathways to HE patterns 1a, 1b and 1c.

mcaa032_suppl_Supplementary_Data_Material_s1

mcaa032_suppl_Supplementary_Data_Material_s2

mcaa032_suppl_Supplementary_Data_Material_s3

mcaa032_suppl_Supplementary_Data_Material_s4

mcaa032_suppl_Supplementary_Data_Material_s5

## FUNDING

This work was supported by the Directorate-general Development Cooperation and Humanitarian Aid (‘Developing climate-smart bananas for the African Great Lakes region’), financed by the Belgian Development Cooperation and by donors through their contributions to the CGIAR Fund, and in particular to the CGIAR Research Program, Roots, Tubers and Bananas. The funders had no role in study design, data collection and analysis, interpretation of results, decision to publish, or preparation of the manuscript.
