# Supplementary figures and images for: Unravelling the complex story of intergenomic recombination in ABB allotriploid bananas
Source: Ann Bot. 2020 Apr 7;127(1):7–20. doi: 10.1093/aob/mcaa032 (PMC7750727; doi:10.1093/aob/mcaa032)

## Slide 1
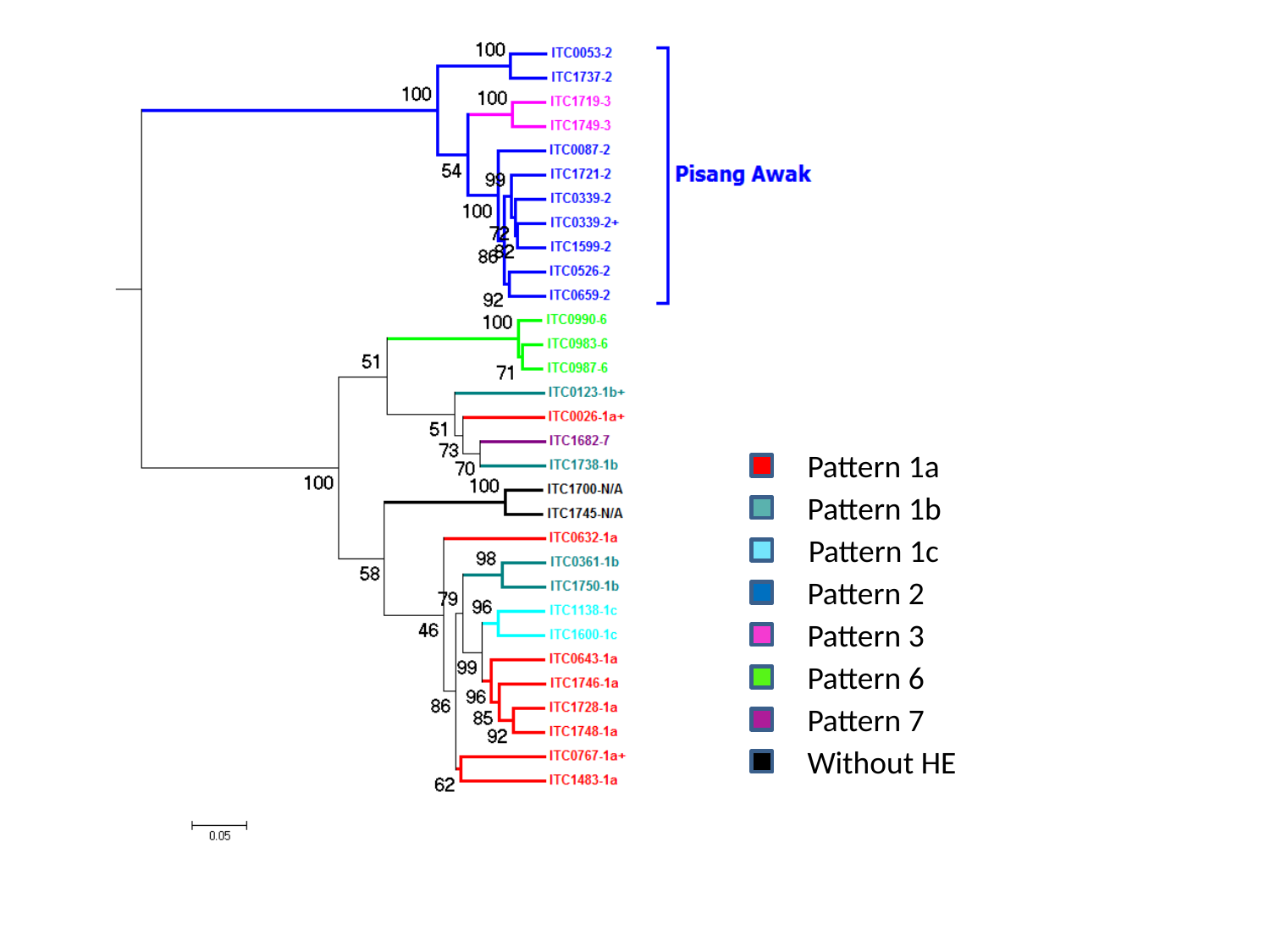

Pattern 1a
Pattern 1b
Pattern 1c
Pattern 2
Pattern 3
Pattern 6
Pattern 7
Without HE

Supplement: mcaa032_suppl_Supplementary_Data_Material_s2 [file mcaa032_suppl_supplementary_data_material_s2.pptx]

## Slide 1
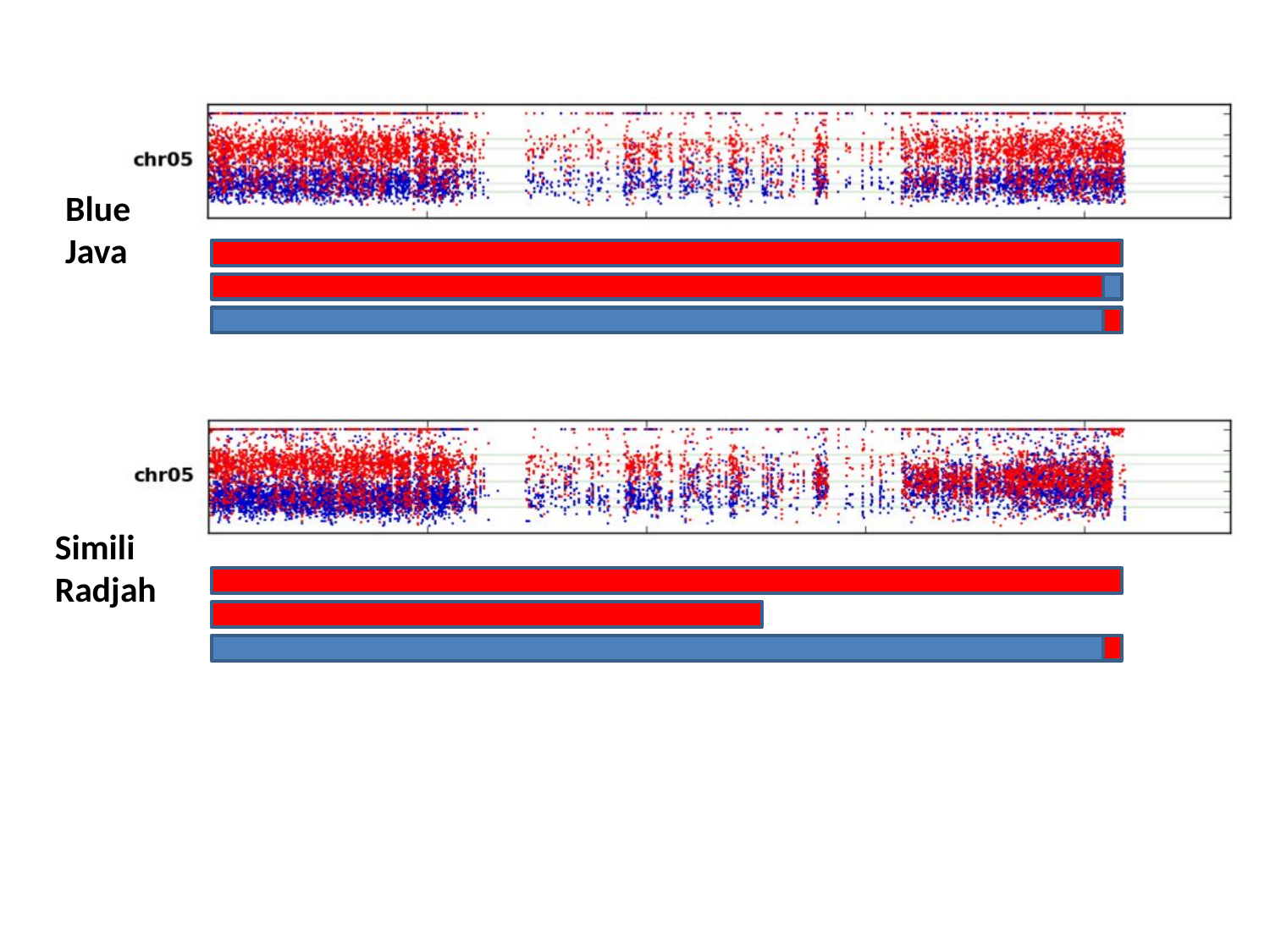

Blue Java
Simili Radjah

Supplement: mcaa032_suppl_Supplementary_Data_Material_s4 [file mcaa032_suppl_supplementary_data_material_s4.pptx]
