# Supplementary material for: Unravelling the complex story of intergenomic recombination in ABB allotriploid bananas
Source: Ann Bot. 2020 Apr 7;127(1):7–20. doi: 10.1093/aob/mcaa032 (PMC7750727; doi:10.1093/aob/mcaa032)
Supplement: mcaa032_suppl_Supplementary_Data_Material_s3 [file mcaa032_suppl_supplementary_data_material_s3.docx]

| Patterns | Chromomosome | | | | | | | | | | | Total |
| --- | --- | --- | --- | --- | --- | --- | --- | --- | --- | --- | --- | --- |
|  | **1** | **2** | **3** | **4** | **5** | **6** | **7** | **8** | **9** | **10** | **11** |  |
| 2x-1 |  |  |  |  |  | 1 |  |  |  |  |  | **1** |
| 2x-2 |  |  |  |  |  |  |  | 1 | 2 |  |  | **3** |
| 2x-3 |  |  |  | 1 |  |  |  |  | 2 |  |  | **3** |
| 2x-4 |  |  |  |  |  |  |  |  | 2 |  |  | **2** |
| 1a |  |  |  | 3 |  |  |  |  | 2 |  | 2 | **7** |
| 1b |  |  |  | 1 |  |  |  |  | 2 |  | 1 | **4** |
| 1c |  | 1 |  | 2 |  |  |  |  | 3 |  | 2 | **8** |
| 2 |  |  |  | 3 |  | 1 | 1 |  | 1 | 1 | 1 | **8** |
| 3 |  |  |  | 1 | 2 |  | 1 | 1 |  | 1 | 1 | **7** |
| 4 |  |  |  |  |  | 1 | 2 |  | 3 | 1 |  | **7** |
| 5 |  |  | 2 | 1 | 2 | 1 | 1 |  | 4 |  | 1 | **12** |
| 6 |  |  | 1 | 1 |  |  |  |  | 1 |  |  | **3** |
| 7 | 2 |  |  | 1 |  |  |  |  | 1 |  |  | **4** |
| Total | **2** | **1** | **3** | **11*** | **4** | **4** | **5** | **2** | **13*** | **3** | **5*** | **53*** |
