# Supplementary material for: Unravelling the complex story of intergenomic recombination in ABB allotriploid bananas
Source: Ann Bot. 2020 Apr 7;127(1):7–20. doi: 10.1093/aob/mcaa032 (PMC7750727; doi:10.1093/aob/mcaa032)
Supplement: mcaa032_suppl_Supplementary_Data_Material_s5 [file mcaa032_suppl_supplementary_data_material_s5.pptx]

## Slide 1
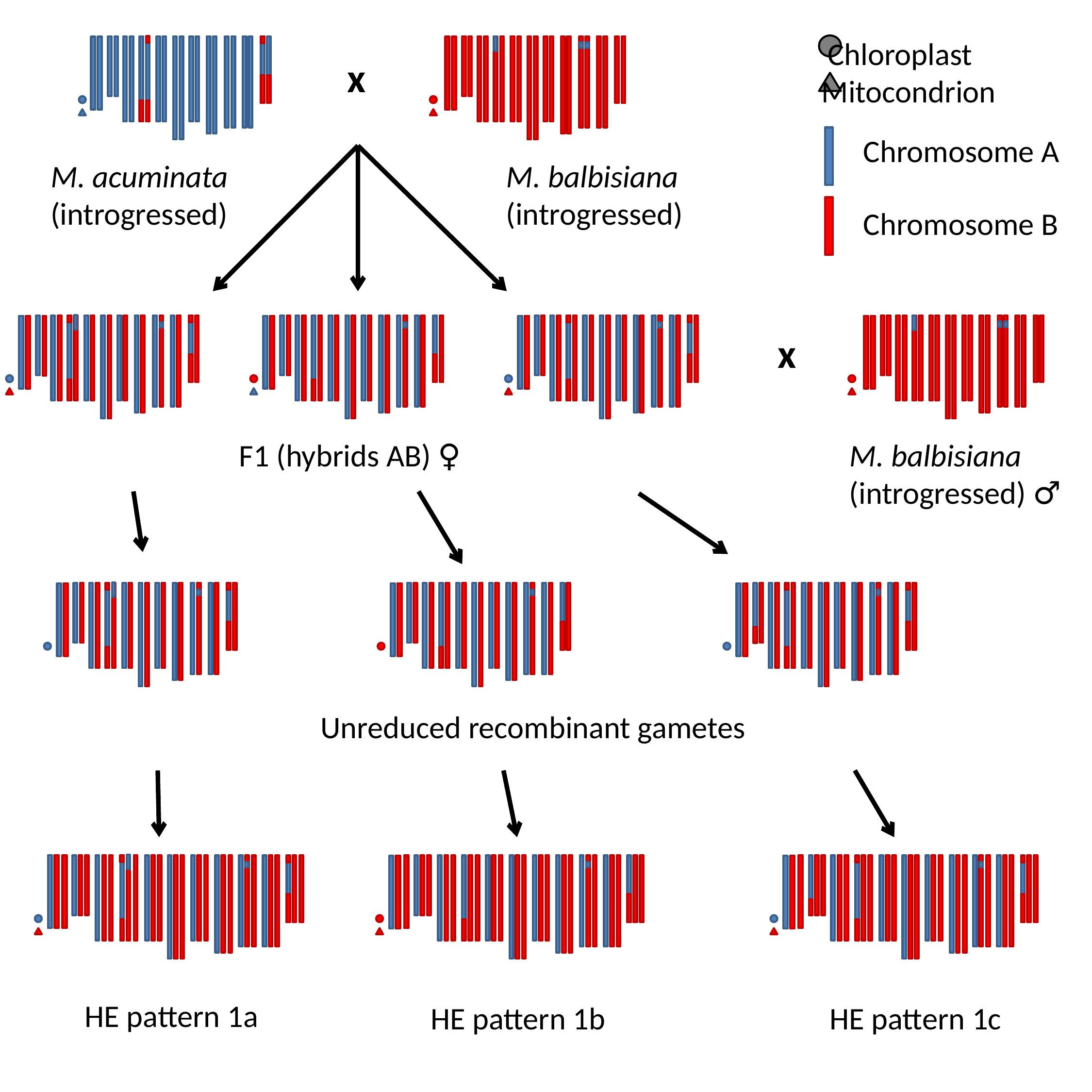

Chloroplast
x
Mitocondrion
Chromosome A
M. acuminata
(introgressed)
M. balbisiana
(introgressed)
Chromosome B
x
F1 (hybrids AB) ♀
M. balbisiana
(introgressed) ♂
Unreduced recombinant gametes
HE pattern 1a
HE pattern 1b
HE pattern 1c
